# Postoperative pain and bleeding after adenotonsillectomy versus adenotonsillotomy in pediatric obstructive sleep apnea: an RCT

**DOI:** 10.1007/s00405-019-05571-w

**Published:** 2019-08-03

**Authors:** Anna Borgström, Pia Nerfeldt, Danielle Friberg

**Affiliations:** 1 Hägersten, Sweden; 2 Department of Clinical Science, Intervention and Technology, CLINTEC, Karolinska Institutet, Stockholm, Sweden; 3 Department of Otorhinolaryngology, Karolinska University Hospital, CLINTEC, Karolinska Institutet, Stockholm, Sweden; 4 Department of Surgical Sciences, Otorhinolaryngology and Head and Neck Surgery, Akademiska Hospital, Uppsala University, Uppsala, Sweden

**Keywords:** Paediatric obstructive sleep apnea, Adenotonsillectomy, Adenotonsillotomy, Tonsillectomy, Tonsillotomy

## Abstract

**Purpose:**

Our previous randomized controlled trial (RCT) of children with obstructive sleep apnea (OSA) showed no significant differences between adenotonsillectomy (ATE) and adenotonsillomy (ATE) in improving nocturnal respiration and symptoms after one year. This is the continuous report with the evaluation of postoperative morbidity concerning bleeding and pain.

**Methods:**

A double-blinded RCT including 79 children, aged 2–6 years, with moderate to severe OSA, randomized to either ATE (*n* = 40) or ATT (*n* = 39). From one to ten days postoperatively, parents filled in a logbook with six pain-related outcomes (parent and child grading pain at different levels, days of analgesic use and return to normal diet). Peri- and postoperative bleeding were also registered.

**Results:**

63 patients (80%) returned the logbook. There were significant differences between groups in only two of the six pain-related outcomes in favor of the ATT group; first day when the children graded themselves as pain free (*p* = 0.021, Log Rank Test), and first day the caregiver estimated pain VAS ≤ 5 (*p* = 0.007, Log Rank Test). Two (5%) cases of postoperative bleeding occurred in the ATE group, one of which needed a return to theatre. No case of postoperative bleeding was seen in the ATT group.

**Conclusions:**

The results from this RCT are in line with previous comparative studies between ATT and ATE. Children operated with ATT had significantly less postoperative pain in one-third of the outcomes, and less bleeding than ATE. However, as the differences in morbidity between the surgical methods were minor the clinical significance is uncertain.

**Trial registration:**

This study was approved by the Swedish Regional Ethics Board in Stockholm, Sweden (Dnr 2011/925-32 and 2013/2274-32) and registered at ClinicalTrials.gov (Trial registration number NCT01676181).

## Introduction

Tonsil surgery remains one of the most commonly performed surgical procedures in children with more than half a million tonsillectomies performed annually in the US, and in Sweden where this study took place, more than 9000 pediatric tonsil operations were performed in 2013 [1, 2]. The predominant indication for all tonsil surgery in children is sleep-disordered breathing/obstructive sleep apnea (OSA) due to tonsil hypertrophy [3]. Traditionally tonsillectomy (TE) with complete removal of the palatine tonsils has been performed. However, TE is associated with high and prolonged postoperative pain and risk of postoperative bleeding. Due to this significant morbidity, several other surgical approaches have been tried over the years, aiming to improve the postoperative recovery. One of these alternative methods is tonsillotomy (TT), with partial removal of the tonsils. TT has become more and more popular over the past decades, and is now the recommended method in obstructive cases in for example Austria [4], and since 2011 TT is more common than TE as method for tonsil surgery in children in Sweden [5] The advantage of TT is that, with its less invasive character, it is considered to be associated with less postoperative pain and a reduced risk of postoperative hemorrhage compared to TE [6–8]. Most of the previous comparative studies have not been double-blinded and none have compared cold steel tonsillectomy with coblation tonsillotomy, the two most common techniques for tonsil surgery in Sweden today [9].

The present study is a continuous report for the same study population as in a recently published study where polysomnographic outcomes one year after adenotonsillectomy (ATE) versus adenotonsillotomy (ATT) were evaluated [10].

The objective of this prospective, randomized double-blinded study was to evaluate and compare secondary outcomes such as postoperative morbidity concerning pain and bleeding after ATE and ATT in a pediatric population.

## Materials and methods

### Study design and population

**Fig. 1:**
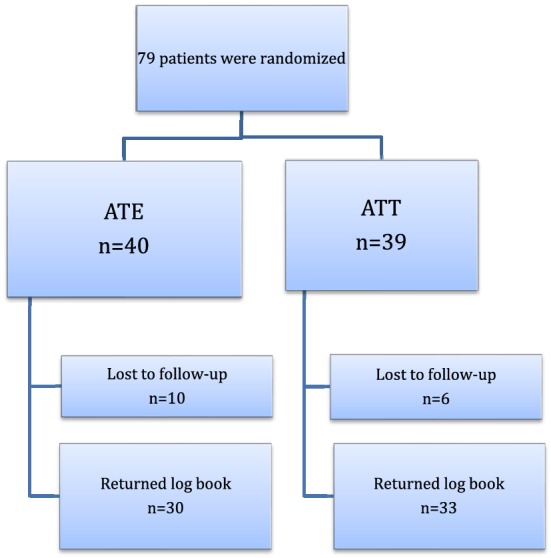
Flow of participants

This study analyzed data from a randomized, parallel group, double-blinded trial conducted at Karolinska University Hospital in Stockholm, Sweden, with enrollment of study patients between November 2011 and April 2015. The study included children 2–6 years old with OSA [apnea–hypopnea index (AHI) 5–30], tonsil hypertrophy 3–4 according to Brodsky [11], non-obese (*z* score > 1.67) and with no co-morbidities. Study participants were randomly assigned to one of two intervention groups; adenotonsillectomy (ATE) or adenotonsillotomy (ATT). The trial was originally designed to evaluate the primary outcome polysomnographic Apnea–Hypopnea Index and this has recently been presented [10]. In the present study, data concerning postoperative morbidity were evaluated and compared between the two groups. Figure 1 shows the flow of participants.

### Randomization and blinding

Randomization took place in the operating room (OR), where the surgeon opened a sealed envelope, randomly assigning study patients to one of the two intervention groups (ATE or ATT). 90 sealed envelopes were made before the study started, 45 for ATE and 45 for ATT, giving a 1:1 allocation ratio. No one but the surgeon and the staff in the OR knew which surgical method was performed. The surgeon did not meet the patients or parents after the operation, they were discharged by another doctor the day after surgery. Thus patients and care providers were double-blinded for intervention method, as was the researchers when analyzing the data.

### Intervention

ATT was performed with coblation® (cold ablation) technique, with partial intracapsular removal of tonsil tissue until remaining tonsils reached the plane between the anterior and posterior tonsillar pillars, whereas in the ATE-group tonsils were bluntly extracapsulary dissected with cold steel technique. In both groups, adenoidectomy with cold steel (ring knife) was performed in the same session. Perioperative hemostasis was obtained with compression, bipolar diathermia or with the coblation device. Perioperative blood loss was documented by the surgeon in the OR (operation room).

### Pain treatment

At discharge the day after surgery, caregivers were given a schedule for analgesics: for children < 17 kg ibuprofen 20–35 mg/kg/day, for children ≥ 17 kg diclofenak 2–3 mg/kg/day, and for all children paracetamol 80–100 mg/kg/day on day 1–3 after surgery and 65–75 mg/kg/day from day 4 and onward. Total treatment time was recommended to be as long as the child had signs of pain. No restrictions for food intake were given.

### Pain registration

**Fig. 2:**
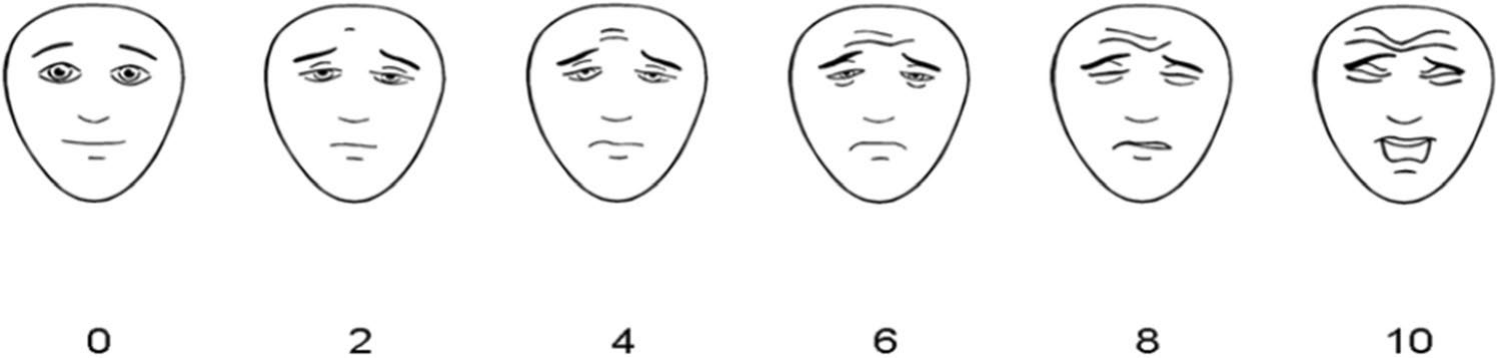
Faces Pain Scale-Revised (FPS-R). https://www.iasp-pain.org/fpsr. Copyright^©^ 2001, International Association for the Study of Pain®. Reproduced with permission

Pain, analgesics given and food intake were registered in a logbook for each day, altogether ten days after surgery. The children’s pain was assessed three times daily from both children and caregivers. The children used the Faces Pain Scale-Revised (FPS-R), a standardized 6-faces Likert-type self-report pain scale scored 0–10 (Fig. 2), validated for children 4 years and older [12, 13], and recommended by Ped-IMMPACT (Pediatric Initiative on Methods, Measurement and Pain Assessment in Clinical Trials) [14]. This scale has been shown to be a useful tool in the quantification of post-tonsillectomy pain in children [15]. Parents/caregivers were asked to assess their children’s level of pain using a Visual Analogue Scale (VAS), numbered 1–10 (1 = no pain and 10 = maximum pain). Further, the children’s food intake was registered using a three-grade scale in which the parents registered the amount of food (‘less than normal’, ‘normal’ or ‘more than usual’) and the type of food texture (‘liquid’, ‘soft’ or ‘normal’).

### Per-postoperative outcomes

The groups were compared regarding six pain-related outcomes: (1) first day when the child estimated pain free (FPS-R 0); (2) first day when the caregiver estimated the child to be pain free (VAS 1); (3) first day when the child estimated pain FPS-R < 6; (4) first day when the caregiver estimated the child’s pain as VAS ≤ 5; (5) first day with no analgesic use; and (6) first day with normal diet (both normal amount of food (‘normal’ or ‘more than usual’) and normal type of food texture).

Further, we evaluated two bleeding variables: (1) perioperative blood loss; (2) cases of postoperative bleeding.

### Statistical analysis

The outcomes 1–6, were determined using Kaplan–Meier analysis, with time-to-event, where event were defined as “free from pain”(child’s + caregiver’s report), “reduced pain”(child’s + caregiver’s report), “no analgesics needed” or “normal diet”. Log-Rank tests were used for comparison between groups.

Perioperative blood loss in milliliter was analyzed by t-test for independent samples.

The difference in cases of postoperative bleeding was analyzed using Fisher’s exact test.

Data were analyzed with IBM SPSS Statistics 20.

## Results

Seventy-nine patients (53 boys and 26 girls) were included and underwent surgical intervention, 40 in the ATE group and 39 in the ATT group, baseline characteristics were comparable for the two groups and are presented in Table 1. Fifty-one (65%) of the children were < 4 years old at the time of surgery.

**Table 1:** Baseline characteristics

	ATE	ATT	*p* value*
*N*	40	39	
Female gender *n* (%)	11 (27%)	15 (38%)	0.30
Age at surgery (months)	47 ± 15	45 ± 15	0.38
Length (cm)	98 ± 13	99 ± 10	0.99
Weight (kg)	15.7 ± 3.1	15.3 ± 3.3	0.99
Tonsil size (1–4)	3.3 ± 0.6	3.5 ± 0.6	0.13

Values are mean ± SD, except for the category gender

*Mann–Whitney *U* test

For the pain-related outcomes (Table 2), 63 patients (80%) returned the logbook, 30 (75%) in the ATE group and 33 (85%) in the ATT group. All patients were included in the analysis of the bleeding parameters (Table 3).

**Table 2:** Pain-related outcomes for adenotonsillectomy (ATE) versus adenotonsillotomy (ATT)

	*n*	ATE	*n*	ATT	
First day when child estimates pain 0 on FPS-R	28	8 (5–9.7)	30	5 (3–8)	*
First day when caregiver estimates pain 1 on VAS	30	8 (5–9.2)	33	6 (3–9)	ns
First day when child estimates pain < 6 on FPS-R	28	2 (1–6)	30	0 (0–1)	ns
First day when caregiver estimates pain ≤ 5 on VAS	30	1 (0–4.5)	33	0 (0–1)	*
First day with no analgetic use	31	8 (7 to > 10)	33	7 (5–9)	ns
First day with return to normal diet	30	7 (5–9)	33	6 (4–8)	ns

Values are median(interquartile range)

*Significant difference (*p* < 0.05, Log-Rank Test)

*ns* non-significant, *FPS-R* Faces Pain Scale—Revised, *VAS* visual analogue scale

### Perioperative blood loss

Perioperative blood loss in the OR was significantly higher in the ATE group; (mean ± SD) 55.1 ± 33.9 ml vs 28.6 ± 15.6 ml in the ATT group, *p* < 0.001 (Table 3).

**Table 3:** Peri- and postoperative data of bleeding for adenotonsillectomy (ATE) versus adenotonsillotomy (ATT)

	*n*	ATE	*n*	ATT	*p*
Perioperative blood loss, ml, mean (SD)	39	55.1 ± 33.9	39	28.6 ± 15.6	**< 0.001** ^a^
Postoperative bleeding n (%)	40	2 (5%)	39	0 (0%)	0.494^b^

Significant difference (*p* < 0.05) shown in bold type

^a^*t* test for independent samples

^b^Fisher’s exact test

### Postoperative pain-related morbidity

**Fig. 3:**
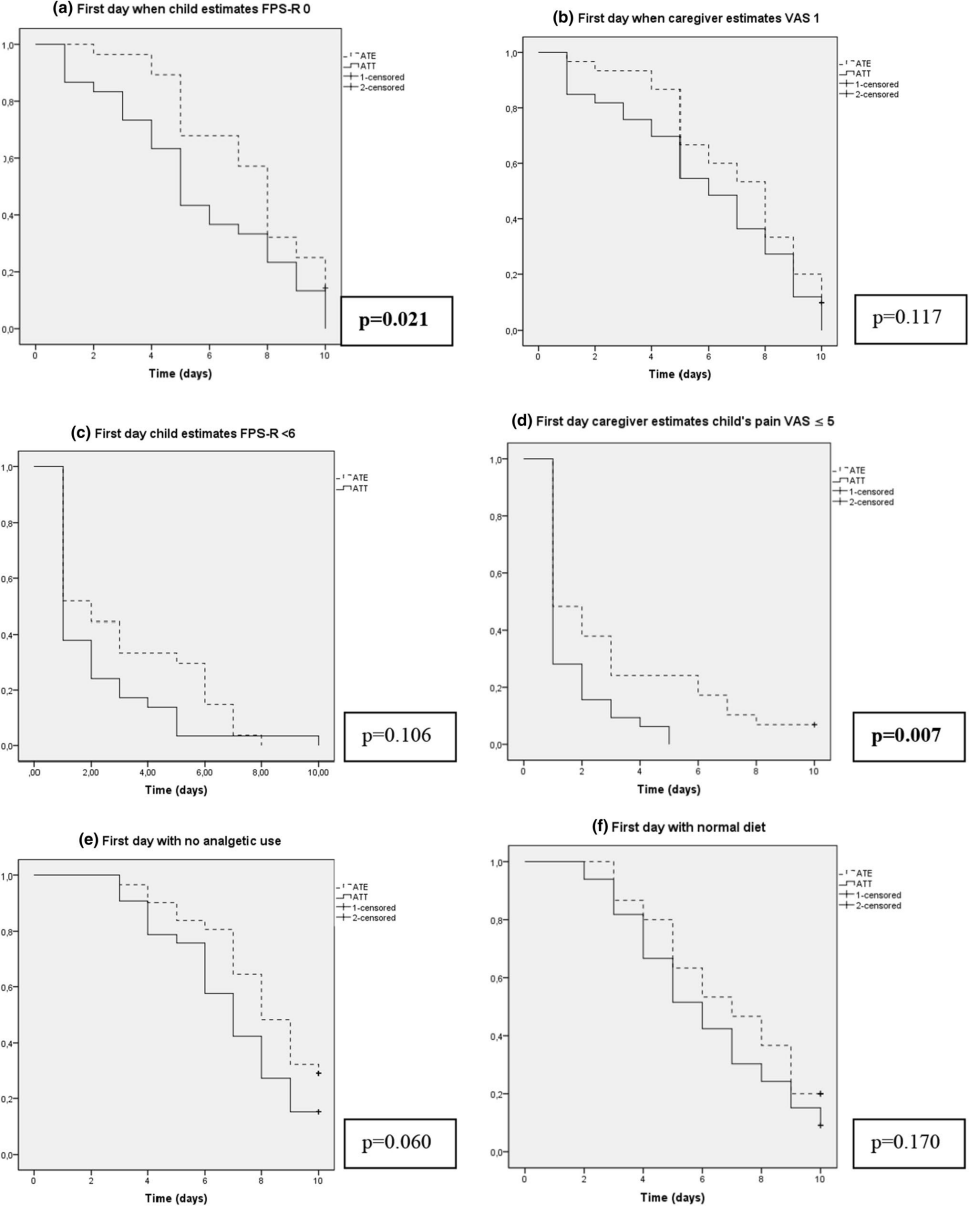
**a**–**f** Log-Rank comparison of Kaplan Meier estimator for postoperative recovery for adenotonsillectomy (*ATE* dotted line) and andenotensillotomy (*ATT* solid line), with estimates of time (days) to pain-related outcomes. For all figures, the y-axis represents the proportion of the patients who are estimated as pain free (**a** + **b**), in reduced pain (**c** + **d**), no longer in need of analgetics (**e**) and returned to normal diet (**f**), at a given time. Patients were censored after 10 days since the log books contained no information thereafter. Two outcomes showed significantly lower pain in the ATT group compared to ATE (**a**, **d**)

Two of the six pain-related outcomes showed significant differences between surgical technique (ATE compared to ATT); the first day when the children graded themselves as pain free (FPS-R 0), which occurred at day median (interquartile range) 8 (5–10) in the ATE-group and day 5 (3–8) in the ATT group, *p* = 0.021(Log Rank Test), and also the first day the caregiver estimated the child´s pain reduced to VAS ≤ 5: 1 (0–4.5) after ATE and 0 (0–1) after ATT, *p* = 0.007 (Log Rank Test). For the other pain-related outcomes no significant differences between the groups were shown. Kaplan–Meier curves and *p* values from Log Rank Tests for pain outcomes are presented in Fig. 3.

### Postoperative bleeding

Postoperative bleeding occurred in two cases (5%) in the ATE group, one within 24 h after surgery needed repeated surgery, whereas the other was readmitted for 24 h observation 7 days after surgery, but needed no surgical intervention. In the ATT group, there was no case of postoperative hemorrhage. The difference between groups was not significant (*p* = 0.494), Table 3.

## Discussion

The main findings in the present randomized double-blinded study are in line with previous comparative studies, which have shown the advantages of TT compared with TE concerning postoperative pain and bleeding.

### Pain

The results suggest that ATT is associated with less postoperative pain than ATE, but the differences were relatively small. Only two of the six pain-related outcomes showed significant differences between the groups: The first day when the child reported being pain free were day median (IQR) 8 (5–10) after ATE and day 5 (3–8) after ATT, and the first day caregiver reported reduction of the child’s pain to VAS ≤ 5 was day median (IQR) 1(1–4.5) for ATE and day 0 (1–2) for ATT. Other pain-related variables showed no significant differences between the groups in this small sample.

Comparable results with our study were found in a review by Walton et al. [14], evaluating TT versus TE in pediatric populations, concluding that TT was equivalent or superior to TE regarding recovery-related outcomes. Only some of the studies in this review were blinded, and none of the blinded studies compared cold steel tonsillectomy with coblation TT, as in the present study. However, one study [17] from the review [14] showed similar results as ours with a randomized, double-blinded study. They compared three different surgical techniques: electrocautery TE, coblation TT, and microdebrider TT, which showed better recovery after TT (coblation and microdebrider were comparable) than after TE in terms of days with pain and return to a normal diet, but with no differences in average pain scores [17].

Moreover, Lister et al. performed a randomized blinded study in which the tonsil on one side was removed with electrosurgical TE, and the other side with microdebrider TT in the same individual, with significantly less pain on the TT side until postoperative day 10 [16]. A proposed explanation to the reduced pain after TT is the preservation of tonsil tissue and the tonsillar capsule, with preserved protection of underlying vessels and nerves. Further, a correlation of the inflammatory response and the extent of the surgical intervention has been suggested, however, a randomized trial evaluating the inflammatory response to surgery after TT vs TE in children did not show any significant differences [19].

The surgical depth of TT tends to vary in previous studies, TTs were often performed all the way to the tonsillar capsule, which is deeper than in our study where TTs were less invasive, stopping at the level of the anterior and posterior pillars. Possibly, the depth of TT could be another factor correlating to the level of postoperative pain. Also, the technique for TT and TE varies in previous studies, making direct comparisons to our results difficult. For example, a randomized study compared cold technique TT (scalpel and scissors) with cold steel TE in a pediatric population, showing less postoperative analgesic use in the TT group, but no differences in pain scoring [20].

Our results demonstrate smaller group differences in pain-related outcomes than some previous non-blinded randomized studies [6, 21]. This discrepancy to our double-blinded study could suggest a possible factor of expectation: the patient’s and parent’s expectation that the pain might be less after ATT than after ATE, depending on the preoperative information given.

Another possible explanation of the smaller group differences is that all the TEs in our study were performed with cold steel technique. A large Swedish study of > 18,000 children 1–18 years undergoing TE, reported less postoperative pain if TE was performed with cold technique than with hot technique [16]. The study also reported a correlation to age, with more pain for children of older ages [22] and all children in our study were very young (mean age of 47 ± 15 months). However, a recent review comparing TE vs TT concluded that neither the extent of surgery or surgical technique seemed to affect the recovery outcomes [23]. The study included 16 RCTs showing only moderate advantages for TT. Nonetheless, we consider our conservative method for TT safe, but as always, the risk of re-growth of the tonsils must be taken into consideration. This risk is highest for the youngest children, especially those under 3 years [24].

### Bleeding

A significant difference in perioperative blood loss was seen, with higher values in the ATE group, but this can be considered of little clinical relevance since the bleeding volumes were relatively small in both groups; 55.1 ± 33.9 ml for TE and 28.6 ± 15.6 ml for TT.

Two cases (5%) of postoperative hemorrhage occurred in the ATE group, one (2.5%) primary (within 24 h) with a return to theatre and one secondary (7 days after surgery). No case of postoperative bleeding was reported in the ATT group.

In comparison, another recent study compared TT and TE in children with OSA reported a postoperative bleeding frequency of 0.2% after TT and 2.9% after TE, of which 1.6% needed a return to theatre [25].

Moreover, the aforementioned Swedish study by Elinder et al. of TE in > 18,000 children, reported a primary bleeding frequency of 2.0% for children undergoing tonsillectomy on OSA indication, with increased risk at older ages [22].

The present study showed no significant difference between the groups regarding postoperative bleeding, which may be due to the small sample size.

### Strengths and limitations

The primary strength of the present study is the randomized, double-blinded design, minimizing the risk of selection bias as well as interpreter- and expectation bias. Moreover, the young age of the children in the study, 2–6 years, is another strength since these are the age groups where tonsil surgery incidence peaks and, therefore, of high clinical interest. Only a few previous studies have included children as young as 2 years.

However, the young age of the included children is also a weakness of the study, as there is no validated tool for assessing pain in children younger than four years, which was the case for 65% of the study population. We used the FPS-R, validated for children 4 years and older [13], and this was chosen since it is easy to use for both parents and children and a recommended tool to assess acute and postoperative pain in children [14]. There are many sources of bias in children’s self-report of pain, e.g., children under five years have a tendency to use only the extremes of scales [26]. It cannot be ruled out that this could have affected the results. On the other hand, returning to normal diet is probably independent of the child’s age, and this parameter did not show significant differences between the groups.

Another weakness is that TT can be performed with several techniques and this study analyzed only a conservative ATT performed with coblation and, therefore, the generalizability can be limited. We chose coblation since it is the most common method for TT in Sweden [27]. Further, the relatively small study population and that no power analysis was performed fort these secondary outcomes. Thus, there is a risk that the study was underpowered to investigate smaller differences between groups. In summary, we consider our results, with the minor differences between techniques, to be of uncertain clinical significance.

## Conclusion

The results from this RCT are in line with previous comparative studies between ATT and ATE. Children operated with ATT had significantly less postoperative pain in one-third of the outcomes, and less bleeding than ATE. However, as the differences in morbitiy between the surgical methods were minor the clinical significance is uncertain.
